# Deciphering the role of miRNA-mRNA interactions in cerebral vasospasm post intracranial hemorrhage

**DOI:** 10.3389/fmolb.2025.1492729

**Published:** 2025-02-06

**Authors:** Xiang Chu, Xiyan Zhu, Honghao Xu, Wenbing Zhao, Debin Guo, Xing Chen, Jinze Wu, Lei Li, Hao Wang, Jun Fei

**Affiliations:** ^1^ Cognitive Development and Learning and Memory Disorders Translational Medicine Laboratory, Children’s Hospital, Chongqing Medical University, Chongqing, China; ^2^ Emergency Department, Daping Hospital, Army Medical University, Chongqing, China; ^3^ Department of Military Traffic Injury Prevention and Control, Daping Hospital, Army Medical University, Chongqing, China; ^4^ Department of Army Occupational Disease, Daping Hospital, Army Medical University, Chongqing, China; ^5^ Trauma Medical Center, Daping Hospital, Army Medical University, Chongqing, China; ^6^ Neurosurgery Department, Daping Hospital, Army Medical University, Chongqing, China

**Keywords:** cerebral vasospasm, intracranial hemorrhage, MiRNA-mRNA regulatory network, bioinformatics analysis, biomarkers, pathogenesis

## Abstract

Cerebral vasospasm (CVS), a serious complication following subarachnoid hemorrhage, is associated with high rates of mortality and disability. Emerging evidence suggests that abnormal miRNA and mRNA are involved in the development of CVS. This study aims to identify essential miRNA-mRNA regulatory pairs that contribute to CVS pathogenesis. We compared the differences between spasm and non-spasm groups after cerebral hemorrhage, identifying 183 differentially expressed genes (DEGs) and 19 differentially expressed miRNAs (DEMs) related to cerebral vasospasm from the GEO database. Further functional enrichment and KEGG analysis revealed that these DEGs were enriched in several terms and pathways, including the PI3K/AKT/mTOR signaling pathway, oxidative phosphorylation pathway, RNA degradation, and folate biosynthesis signaling pathway. By employing the degree scores method for each gene, we identified the top 10 genes and developed a protein-protein interaction (PPI) network. Additionally, we discovered 19 DEMs associated with CVS and integrated them with mRNA dataset analysis to construct a miRNA-mRNA network, which comprised 8 functionally differentially expressed DEMs and 6 target mRNAs. Experimental validation confirmed the significant regulatory roles of four miRNAs (Let-7a-5p, miR-24-3p, miR-29-3p, and miR-132-3p) and two mRNAs (CDK6 and SLC2A1) in the pathogenesis of CVS. In conclusion, this comprehensive study identifies pivotal miRNAs and their target mRNAs associated with CVS through an integrated bioinformatics analysis of miRNA-mRNA co-expression networks. This approach elucidates the intricate molecular mechanisms underlying CVS and uncovers potential therapeutic targets, thereby providing a valuable foundation for refining and optimizing future treatment strategies.

## Highlights

1. Identification of 183 DEGs and 19 DEMs.
2. Construction of miRNA-mRNA network.
3. Four miRNAs and two mRNAs were validated.
4. Potential therapeutic targets identified.

## Introduction

Intracranial hemorrhage (ICH) is a prevalent cerebrovascular disease and a critical medical emergency (Zhang et al., 2024). Approximately 75%–80% of subarachnoid hemorrhage (SAH) cases result from ruptured intracranial aneurysms, predominantly affecting individuals aged 40 to 60. Cerebral vasospasm (CVS), one of the most severe complications, occurs in up to 70% of cases, typically manifesting within days to 2 weeks after SAH (Treggiari-Venzi et al., 2001). CVS-induced cerebral ischemia, delayed ischemic neurological deficits (DIND), cerebral infarction, and fatalities significantly contribute to the high mortality and disability rates associated with this disease (Zarrin et al., 2024). Therefore, promptly identifying, preventing, and managing CVS in individuals with cerebral hemorrhage is crucial to minimize brain tissue damage and enhance patient outcomes.

CVS generally occurs in the brainstem or major arteries, characterized by localized, segmental, or diffuse spastic manifestations due to ultrastructural and functional changes in endothelial cells and smooth muscle cells. The resulting vasoconstriction and impaired vasodilation lead to delayed stenosis and narrowing of the vascular lumen, reducing cerebral perfusion pressure and blood flow and ultimately causing cerebral infarction (Harrod et al., 2005; Macdonald, 2014). The precise etiologies of CVS are complex and multifactorial. Factors such as red blood cells, metabolic byproducts, and bloody cerebrospinal fluid within blood clots can act as mechanical stimulants on intracranial vessels, enhancing vasoconstrictive function and leading to CVS. Oxyhemoglobin is critical in triggering CVS by generating oxygen free radicals through lipid peroxidation, resulting in serious tissue cell membrane impairment (Dietrich and Dacey, 2000; Ishiguro et al., 2006; Yang et al., 2019). This oxidative process increases endothelin (ET) production and disrupts vascular relaxation by interacting with nitric oxide (Zhang et al., 2001). Additionally, the inflammatory response initiated by the rupture of an intracranial aneurysm results in blood accumulation in the subarachnoid space and brain ventricles. This accumulation leads to endothelial cell apoptosis and decreased endothelial cell function, ultimately contributing to CVS development. Research by Aihara and Kasuya has shown that expression levels of IL-6, IL-1α, and Intercellular Adhesion Molecule-1 (ICAM-1) following intracerebral hemorrhage correlate with the onset of CVS (Aihara et al., 2001).

Brain vascular endothelial and smooth muscle cells play crucial roles in the development and progression of CVS. Following cerebral hemorrhage, arterial remodeling, inflammation, altered extracellular matrix, and cell damage result in prolonged vasoconstriction and impaired vasodilation, leading to vascular spasm. The lysis of red blood cells and arachidonic acid and their metabolic byproducts can damage endothelial cells, release bioactive substances such as endothelin and serotonin, inhibit prostacyclin synthase activity, and promote platelet adhesion and aggregation. This process increases thromboxane A2 release, causing an imbalance between vasoconstriction and vasodilation, promoting cerebrovascular spasm (Cahill et al., 2006; Viderman et al., 2023). Activation of Ca2+ channels on smooth muscle cell membranes further contributes to vasoconstriction. Oxyhemoglobin induces endothelin-1 secretion by smooth muscle cells via the NF-κB and Rho kinase/PKC pathways, leading to vasoconstriction (Peeyush Kumar et al., 2019).

Additionally, oxyhemoglobin can increase and activate voltage-gated calcium channels in cerebral arterial smooth muscle cells while inhibiting voltage-gated potassium channels, leading to a significant influx of Ca2+ (Ishiguro et al., 2006). The phosphorylation of calcium-dependent myosin light chain kinase causes contraction of smooth muscle cells, representing the classical theory of vasoconstriction. Various factors such as inflammation, endothelial cell apoptosis, oxidative stress response, NO signaling pathway, and cell membrane dysfunction contribute to the development of CVS, complicating the identification of relevant biomarkers for accurate prediction and diagnosis, ultimately impacting patient outcomes. Thus, extensive identification of underlying biomolecules and key pathways related to CVS pathophysiological mechanisms is urgently needed.

Microarray and high-throughput sequencing, combined with integrated bioinformatics analysis, have significantly advanced the identification of novel genes and pathways involved in disease pathology. Studies have examined changes in gene expression and miRNA levels in fluid systems such as blood and cerebrospinal fluid as potential biomarkers for CVS following intracerebral hemorrhage. Antoine Baumann et al. demonstrated that NRG1 and HTRA1 impact the proliferation and migration of vascular smooth muscle cells, contributing to CVS development (Baumann et al., 2013). Stanley S. discovered that miR-27a-3p, miR-516a-5p, miR-566, and miR-1197 are significantly upregulated in the vasospasm group through cerebrospinal fluid (CSF) miRNA sequencing, suggesting these miRNAs could serve as CSF biomarkers predisposing individuals to CVS (Stylli et al., 2017). Additionally, elevated expression levels of miR-15a (Kikkawa et al., 2017), miR-24 (Li et al., 2018), and miR-195-5p (Tsai et al., 2021) in SAH patients are closely associated with CVS onset. miR-199a, miR-497, and miR-365 target TGF-β and MAPK signaling, influencing inflammation, matrix degradation, vascular smooth muscle cell apoptosis, and vessel wall rupture (Supriya et al., 2022). Despite studies on genes with DEGs and DEMs, significant individual variability among patients has hindered clinical validation, resulting in discrepancies in using these mRNAs and miRNAs for CVS diagnosis. Furthermore, few studies have conducted integrative analyses of miRNA-mRNA regulatory networks to elucidate their biological functions, necessitating further investigation.

In the present study, mRNA expression profiles (GSE37924) and miRNA expression datasets (GSE165698) from the GEO database were selected to identify DEGs between CVS and non-CVS groups. Potential pathological mechanisms of CVS were explored through functional enrichment analysis, including Gene Ontology (GO) and Kyoto Encyclopedia of Genes and Genomes (KEGG), and a protein-protein interaction (PPI) network was constructed. Furthermore, a miRNA-mRNA regulatory network was developed to investigate the roles of key miRNAs and their target genes in cultured cells and brain tissues affected by SAH. This approach facilitates a comprehensive understanding of molecular mechanisms and aids in discovering potential diagnostic biomarkers or therapeutic targets for CVS.

## Methods

### Patients

The research adhered to the principles of the Helsinki Declaration and was approved by the Ethics Committee of Daping Hospital (Ethical Number: 2024–247), Army Medical University. Specimens were collected with informed written consent from patients or their families. Briefly, data and specimens were collected from adult patients aged 18 and above who underwent endovascular treatment for intracranial aneurysm following aneurysmal subarachnoid hemorrhage (aSAH) at Daping Hospital from 2022 to 2023. Inclusion criteria included: 1) definitive diagnosis of subarachnoid hemorrhage with evidence of intracerebral hematoma or intraventricular hemorrhage on CT or MRI; 2) confirmation of vascular spasm by DSA; 3) indication of ischemia on perfusion-weighted MRI, CT angiography (CTA), or CT perfusion imaging with diffuse cerebral edema. Exclusion criteria included a history of prior cranial surgery, brain malignancy, systemic inflammatory disease, chronic use of biologic inflammatory modulators, and absence of signed informed consent. Pre-surgical assessments included detailed medical history, neurological examination, and neuroimaging studies. Microsurgical clipping of the aneurysm and removal of surrounding clot tissue, including intracerebral hematomas, were performed to preserve normal brain tissue and blood flow integrity. All necrotic tissues, clots, and foreign bodies were excised, focusing on edematous tissue from hemorrhagic foci without significant blood infiltration, which served as experimental material. Paraffin sections of tissues from patients who developed postoperative vasospasm underwent immunohistochemical and hematoxylin and eosin (HE) staining.

### Screening of differentially expressed mRNA and miRNA

To identify key genes and miRNAs and construct miRNA-target gene regulatory networks related to CVS, we searched the GEO database using “cerebral vasospasm” as a keyword. We collected one mRNA-sequencing dataset (GSE37924) and one miRNA-sequencing dataset (GSE165698). After comparing the vasospasm and non-vasospasm groups, the Wilcox test was used to assess miRNA expression, selecting candidates with corrected p-values below 0.01. Similarly, the ROC test was employed to calculate the differential expression of mRNAs, identifying candidates with p-values below 0.01. This approach yielded a list of differentially expressed mRNAs and miRNAs.

### Functional enrichment analysis

The GO and KEGG databases were used for biological function analysis to investigate the functional roles of differentially expressed genes (DEGs) in miRNA-mRNA networks. Enrichment values of GO terms and KEGG pathways were determined using the hypergeometric test, with q-values adjusted from p-values. A significance threshold was set at q-value <0.01.

### PPI network construction and module analysis

The identified DEGs were analyzed to construct a PPI network by mapping them to the STRING database, using a combined score threshold of ≥0.4. Cytoscape was employed to visualize the protein interaction network, while CytoHubba was utilized to predict significant nodes or subnetworks based on topological algorithms. Specifically, the Maximal Clique Centrality (MCC) algorithm was applied to identify hub genes associated with CVS.

### WGCNA analysis

Weighted Gene Co-expression Network Analysis (WGCNA) was conducted using the “WGCNA” R package in this study. Key steps included gene filtering, removal of outlier samples, selection of a soft threshold (β), calculating the topological overlap matrix (TOM), module merging, and correlation calculation. During the WGCNA process, important parameters such as minModuleSize were set to 30 and MEDissThres to 0.25. Gene modules related to CVS were identified based on correlation and p-values between sample information and module eigengenes (ME). Key genes associated with CVS were selected based on the following criteria: cor. Gene GS > 0.6 and cor.gene MM > 0.7.

### Screening for miRNAs related to CVS

The miRNA dataset (GSE165698) comprised 136 samples from patients with cerebral hemorrhage, including 56 with CVS, 64 without CVS, and 16 healthy controls. Comparing samples with and without CVS, we identified 19 differentially expressed miRNAs. Among these, 11 miRNAs showed higher expression in the non-vasospasm samples, while 8 miRNAs exhibited higher expression in the vasospasm samples (Supplementary Table S6).

### Identification of the miRNA-mRNA regulatory network

MiRNA target gene prediction was conducted using three databases: TargetScan, miRDB, and miRTarBase. Only genes identified in all three databases were selected as miRNA target genes for further investigation. The regulatory network between the overlapping differentially expressed miRNAs (DEMs) and their target genes was visualized using Cytoscape after processing. To validate the involvement of these miRNAs in CVS, their target genes were intersected with DEGs. Genes that showed a negative association with their corresponding miRNAs were filtered based on this criterion.

### Cell culture and treatment

Primary human cerebral artery smooth muscle cells were obtained from Pricella (Cat. No. CP-H116) and cultured in CM-H116 smooth muscle growth medium (Pricella, Cat. No. CM-H116) under standard conditions: a humidified incubator at 37°C with 5% CO_2_. The CM-H116 medium was supplemented with fetal bovine serum (FBS), growth supplement, penicillin, and streptomycin. Cells were treated with hemin (Sigma, Cat. No. 51280) at a final concentration of 100 µM for varying durations, followed by RNA extraction for qPCR analysis.

### Quantitative real-time PCR analysis

QPCR was validated using the PrimeScript RT reagent Kit and SYBR Premix Ex Taq II (Takara). Relative gene expression was calculated using the 2^−ΔΔCT^ method (ΔCt = Ct miRNA/mRNA - Ct normalizer; Ct: threshold cycle). The primer sequences used in this study were as follows: H-CDK6 (1)-S: TCCCAGGCAGGCTTTTCAT, H-CDK6 (1)-A: GGTCCTGGAAGTATGGGTGAGA; H-EZH2 (1)-S: GATGAAGCTGACAGAAGAGGGAA, H-EZH2 (1)-A: GCATAGCAGTTTGGATTTACCGA; H-RB1-S: CTGTGGATGGAGTATTGGGAGG, H-RB1-A: TTTCCAATTTGCTGAAGAGTGC; H-DHFR(1)-S: GCTGCTGTCATGGTTGGTTC, H-DHFR(1)-A: GAGGTTGTGGTCATTCTCTGGA; H-SLC2A1-S: GCTTCTCCAACTGGACCTCAAA, H-SLC2A1-A: GAAGAACAGAACCAGGAGCACAG; H-GAPDH-S: GGAAGCTTGTCATCAATGGAAATC, H-GAPDH-A: TGATGACCCTTTTGGCTCCC.

The stem-loop reverse transcription method was employed for miRNA detection using a reverse transcription kit (TaKaRa, Cat NO: RR037A), following the same procedures as qPCR. The primer sequences used for miRNA detection were: hsa-let-7a-5p-RT: CTCAACTGGTGTCGTGGAGTCGGCAATTCAGTTGAGAACTATAC, hsa-let-7a-5p-S: ACACTCCAGCTGGGTGAGGTAGTAGGTTGT; hsa-miR-24-3p-RT: CTCAACTGGTGTCGTGGAGTCGGCAATTCAGTTGAGCTGTTCCT, hsa-miR-24-3p-S: ACACTCCAGCTGGGTGGCTCAGTTCAGCAG; hsa-miR-29a-3p-RT: CTCAACTGGTGTCGTGGAGTCGGCAATTCAGTTGAGTAACCGAT, hsa-miR-29a-3p-S: ACACTCCAGCTGGGTAGCACCATCTGAAAT; hsa-miR-132-3p-RT: CTCAACTGGTGTCGTGGAGTCGGCAATTCAGTTGAGCGACCATG, hsa-miR-132-3p-S: ACACTCCAGCTGGGTAACAGTCTACAGCCA; Universal primer: TGGTGTCGTGGAGTCG; U6-S: CTCGCTTCGGCAGCACA, U6-A: AACGCTTCACGAATTTGCGT.

### Immunohistochemical staining

Immunohistochemical analysis of paraffin-embedded brain sections from patients was performed utilizing a microwave in 0.01 mol/L citrated buffer. Endogenous peroxidase activity was neutralized with 0.3% hydrogen peroxide in PBS, followed by blocking with goat serum working solution for 1 h at room temperature. Sections were then incubated overnight at 4°C with primary antibodies. Subsequently, sections were washed with PBS and exposed to fluorochrome-conjugated secondary antibodies. Immunoreactivity was visualized using a confocal microscope (Pannoramic MIDI, 3DHISTECH). The antibodies utilized in this study are detailed in Supplementary Table S8.

### Statistical analysis

Data were analyzed utilizing GraphPad Prism software version 8.3.0 and R language version 3.6.3 (https://cran.r-project.org/). Continuous parametric data are presented as mean ± SD. A one-way analysis of variance (ANOVA) was performed for normally distributed data with homogeneous variance, followed by a *post hoc* Student Newman-Keuls test. A p-value <0.05 was considered statistically significant, denoted as ***p < 0.001, **p < 0.01, and *p < 0.05.

## Results

### ICH induces neuropathological injuries in human brains

This study collected 13 samples of aneurysm tissue and surrounding hematoma from patients with spontaneous subarachnoid hemorrhage between 2022 and 2023, with 6 patients experiencing CVS (Hunt-Hess grades II-IV; Fisher grades II-IV). Supplementary Table S1 summarizes the clinical characteristics of the SAH patients.

**FIGURE 1:**
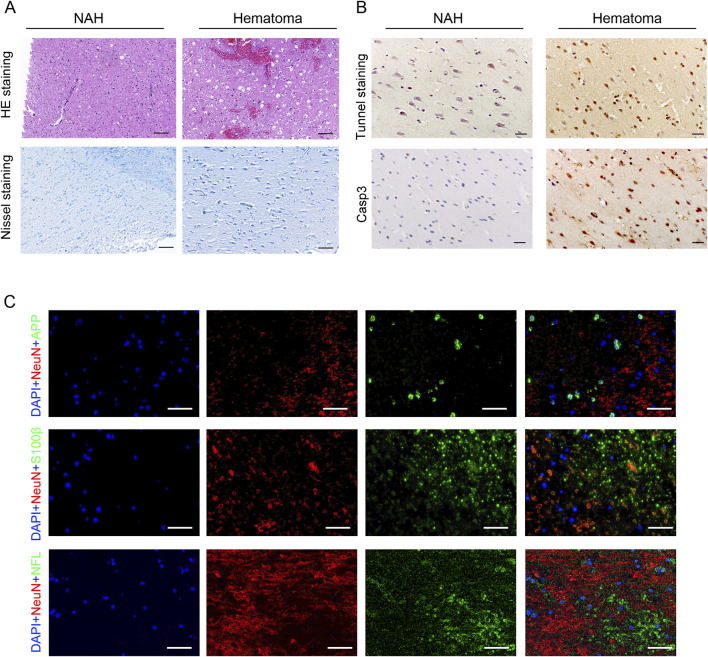
Pathological changes in the brain of patients with ICH. **(A)** Visualization of pathological reactions in patients’ hematomas via HE staining and Nissl staining. Bars, 50 μm. **(B)** Representative TUNEL and Casp3 staining images in the near-adjacent hematomas (NAH) and hematoma areas. Bars, 50 μm. **(C)** Immunostaining hematoma areas from patients with ICH using anti-APP, S100β, NFL, and NeuN antibodies. Nuclei are stained with DAPI. Bars, 50 μm.

Initially, we investigated the pathological responses of hematomas in patients diagnosed with ICH. HE and Nissl staining showed neuron cytoplasm shrinkage, nucleus condensation, and intensified staining, indicating brain tissue damage due to cerebral hemorrhage (Figure 1A). Subsequently, TUNEL and Casp3 staining on brain tissue revealed significant neuronal apoptosis in the hematoma areas (Figure 1B). Previous studies have suggested that neuron-specific enolase (NSE), S-100 calcium-binding protein (S-100), and amyloid precursor protein (APP) could serve as serum biomarkers for brain injury (Adrian et al., 2016; Azizi et al., 2021). We identified increased expression of these proteins in the brain tissues of patients following cerebral hemorrhage (Figure 1C).

### Screening and analysis of differentially expressed mRNA

Subsequently, we analyzed the mRNA expression dataset from GSE37924, comprising 128 samples categorized into three groups: 32 without vasospasm, 32 with vasospasm, and 64 diverse cases. A total of 183 genes showed differential expression when comparing the vasospasm and non-vasospasm groups. Among these, 114 genes exhibited higher expression in the vasospasm group, while 69 genes were upregulated in the non-vasospasm group. The details of DEGs are provided in Supplementary Table S2.

**FIGURE 2:**
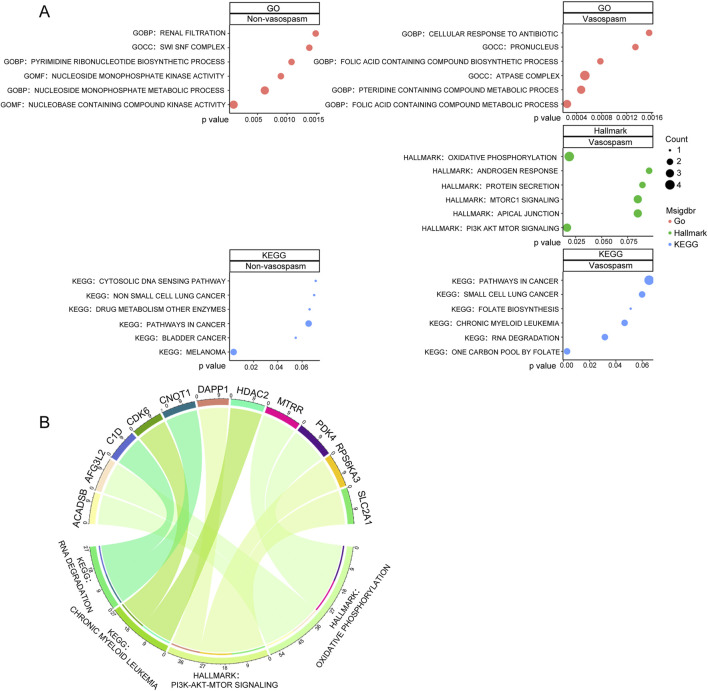
Screening and pathway analysis of DEGs associated with vasospasm. **(A)** GO, HALLMARK, and KEGG enrichment analysis of DEGs in vasospasm and non-vasospasm groups. **(B)** The plot on the left illustrates the correlation between the upregulated genes and their associated pathways in the vasospasm groups; the right plot demonstrates the upregulated genes and their relevant pathways in the non-vasospasm groups.

We conducted GO functional enrichment analysis, Hallmark, and KEGG function enrichment to explore the functions associated with differentially expressed genes (DEGs). GO analysis revealed that DEGs in the vasospasm group were significantly enriched in several GO biological process (GO-BP) categories, including folic acid-containing compound metabolic process, pteridine-containing compound metabolic process, and folic acid-containing compound biosynthetic process. The GO cellular component (GO-CC) category was primarily associated with the ATPase complex and pronucleus. In the GO molecular function (GO-MF) category, significant enrichments were observed in actin binding, integrin binding, potassium channel regulator activity, and nucleobase-containing compound kinase activity (Figure 2; Supplementary Figure S1). The functional phenotypes associated with DEGs in CVS cohorts were notably linked to PI3K/AKT/mTOR signaling and oxidative phosphorylation based on Hallmark enrichment analysis.

KEGG pathway analysis identified significant associations with pathways, including one carbon pool by folate, RNA degradation, chronic myeloid leukemia, and folate biosynthesis within the CVS cohorts (Figure 2A). Detailed information on the top 10 enriched pathways from the GO and KEGG analysis is presented in Supplementary Figure S1. Figure 2B illustrates the relationship between key genes and their associated pathways in vasospasm and non-vasospasm groups. These findings contribute to a deeper understanding of the mechanisms underlying CVS in the pathogenesis of ICH.

### WGCNA analysis of CVS-related genes

In genetics and molecular biology research, gene modules consist of groups of functionally related genes that collaborate to execute specific biological processes. Understanding pathway enrichment within these gene modules is crucial for elucidating the underlying mechanisms and molecular pathways involved in CVS. To explore these correlations, we conducted WGCNA to examine the relationship between gene expression levels and CVS-related pathways.

**FIGURE 3:**
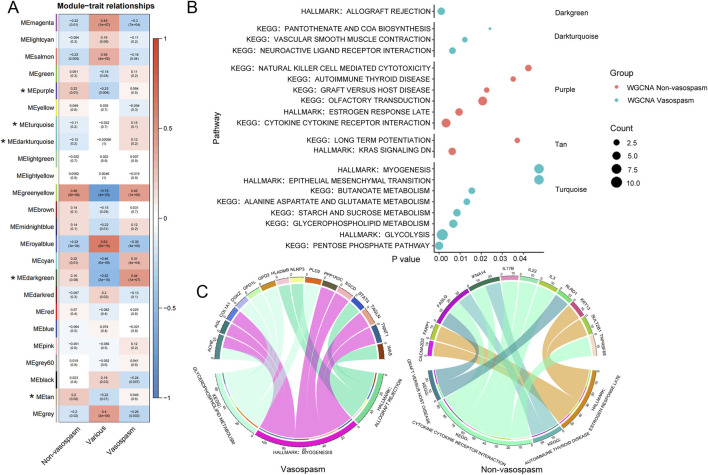
WGCNA analysis of transcriptional modules from peripheral blood RNA-seq data. **(A)** Module-trait heatmap showing the correlation between the obtained gene module and phenotype. The horizontal axis represents phenotypes, and the vertical axis represents gene modules. Positive correlation is indicated by red, and negative correlation by blue. The numbers above indicate a correlation in the boxes; the significance level is below. **(B)** Visualization of gene modules and functional pathways corresponding to differentially expressed genes identified through WGCNA analysis. The *X*-axis represents the significance level of pathway enrichment (P value), while the *Y*-axis represents the corresponding pathways. **(C)** Schematic diagram depicting the interconnection between pivotal genes and their corresponding pathways. The upper section displays the genes, while the lower section presents the corresponding KEGG pathways for those genes. WGCNA, Weighted Gene Co-expression Network Analysis.

The analysis identified 24 modules, each marked with a different color. A heat map of module–trait relationships was generated using the Spearman correlation coefficient to evaluate the association between each module and the disease (Figure 3A; Supplementary Table S3). Three modules (Darkgreen, Dark turquoise, and Turquoise) strongly correlated with CVS and were selected as CVS-related modules. We then predicted the enriched gene pathways of these CVS-associated modules, as shown in Figure 3B. Pathways such as vascular smooth muscle contraction, neuroactive ligand-receptor interaction, pentose phosphate, and glycolysis are involved in vasospasm caused by intracerebral hemorrhage (Supplementary Table S4).

After identifying a close relationship between genes and the occurrence and development of CVS within the three modules, we identified 15 key genes related to CVS based on the filtering conditions of cor. Gene GS > 0.6 and cor. Gene MM > 0.7, including ACHE, AGL, COL1A1, and DGKZ. Pathway analysis was conducted by integrating the core genes from each module with KEGG pathways. The results indicated that pathways such as glycerophospholipid metabolism, myogenesis, and allograft rejection are involved in the pathogenesis of CVS (Figure 3C).

### PPI network construction and module analysis

**FIGURE 4:**
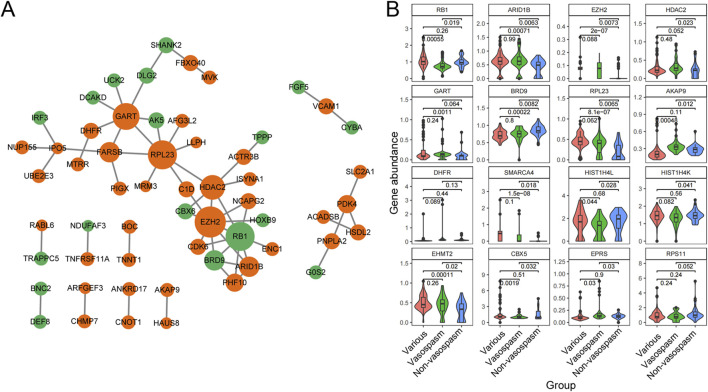
PPI network and Hub gene identification between vasospasm and non-vasospasm groups. **(A)** Construction of a sub-network module with the highest score in the PPI network based on 183 differentially expressed genes between the vasospasm and non-vasospasm groups. Each node in the graph represents a mRNA, and the edges between nodes represent interactions between genes. The orange color indicates genes upregulated in the vasospasm group, while the green color indicates genes upregulated in the non-vasospasm group. **(B)** Expression levels of the top 16 genes between vasospasm and non-vasospasm groups in the GEO dataset GSE37924. PPI, protein-protein interaction, DEGs, differentially expressed genes.

To identify the significant pathways of CVS, we uploaded the filtered 183 DEGs into STRING and exported the interaction data as a TSV file for further network construction in Cytoscape. After constructing the PPI network, we employed the MCC algorithm in CytoHubba to identify 10 hub genes (Figure 4A). Among these, ARID1B, EZH2, HDAC2, GART, IPO5 (RANBP5), RPL23, AKAP9, and DHFR were upregulated in vasospasm groups, while RB1 and BRD9 were upregulated in non-vasospasm groups (Supplementary Table S5). The expression profiles of the top 16 most prominent genes in the vasospasm and non-vasospasm groups are shown in Figure 4B.

### Construction of miRNA-mRNA interactions and regulatory networks

**FIGURE 5:**
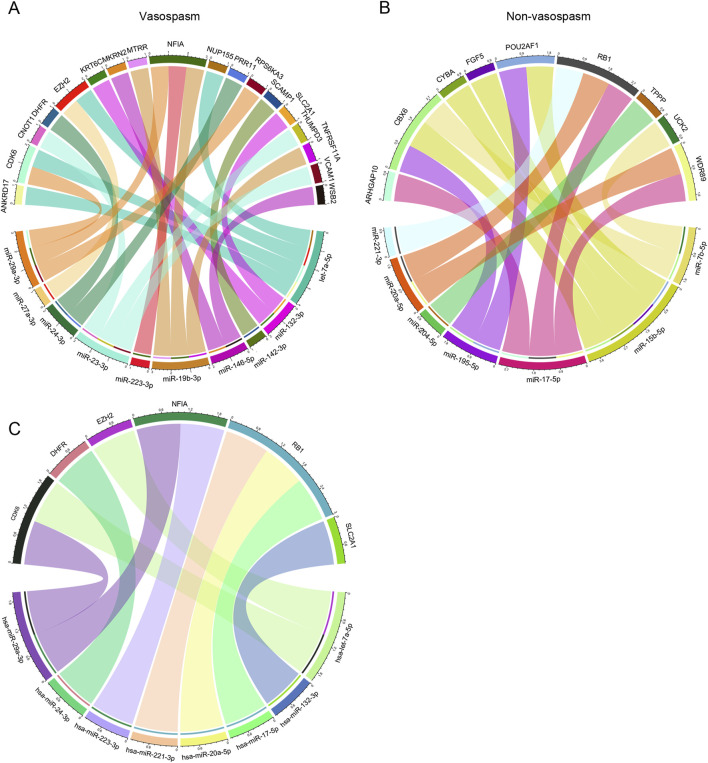
Integrated CVS-related key miRNA-mRNA co-expression regulatory network: **(A, B)** miRNA-mRNA co-expression regulatory network of miRNAs and their target mRNAs in vasospasm and non-vasospasm groups. **(C)** Key miRNA-mRNA regulatory network involved in vasospasm following intracerebral hemorrhage.

Next, we explored the role of miRNA in CVS. The miRNA dataset from GSE165608 comprises 136 samples, including 56 patients diagnosed with delayed cerebral vasospasm (DCV), 64 patients without DCV, and 16 healthy controls. We specifically examined the differences in miRNA profiles between the vasospasm and non-vasospasm groups. Nineteen differentially expressed miRNAs were identified using similar filter criteria as for DEGs (*P* < 0.01) (Supplementary Table S6). Among these, 11 miRNAs were highly expressed in the non-vasospasm group, while 8 were expressed in the vasospasm group. As shown in Figures 5A, B, we identified 15 miRNA-mRNA regulation pairs in the non-vasospasm group, corresponding to 9 genes and 7 miRNAs. In the vasospasm group, we identified 22 pairs corresponding to 18 genes and 10 miRNAs. Based on the criterion of negative regulation between miRNA and mRNA, we further filtered and constructed a miRNA-mRNA correlation network (Figure 5C; Supplementary Table S7). The results showed that 8 miRNAs might regulate the expression of 6 target genes (CDK6, DHFR, EZH2, NFIA, RB1, and SLC2A1). Notably, the RB1 gene and its corresponding 3 miRNAs (hsa-miR-221-3p, hsa-miR-20a-5p, and hsa-miR-17-5p) were associated with the non-vasospasm group, while the other miRNA-mRNA pairs were associated with the vasospasm group. We also predicted and retrieved information on the modes of action of the filtered miRNA-mRNA pairs from databases, revealing that these miRNAs directly regulate the 3′UTR of the mRNAs, which aligns with our analysis (Supplementary Tables S9, S10).

### Validation of the miRNA-mRNA interaction pairs during the vasospasm process

**FIGURE 6:**
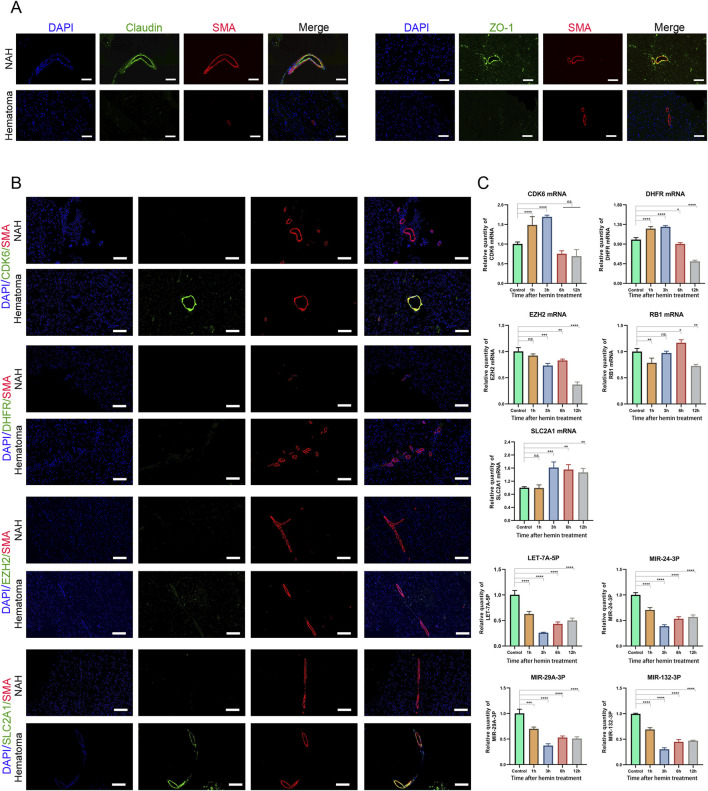
Prediction and validation of miRNAs and their target genes **(A)** Confocal microscopy images of dual immunofluorescence staining in brain sections derived from ICH patients. Claudin and ZO-1 are stained green using an Alexa Fluor 488 conjugated secondary antibody. Vascular smooth muscle cells are counterstained with SMA antibody, stained in red fluorescence, using an Alexa Fluor 555 conjugated secondary antibody. The cell nuclei are stained and labeled with DAPI: bars, 50 μm. **(B)** Immunostaining of NAH and hematoma areas from patients with ICH using anti-CDK6, DHFR, EZH2, and SLC2A1 antibodies. Bars, 200 μm. **(C)** Real-time PCR quantification of mRNA and miRNA levels in human cerebral artery smooth muscle cells after treatment with hemin (100 μM). Data are presented as fold changes relative to the control group, normalized to GAPDH. The data represent at least three independent experiments.

Disrupting the blood-brain barrier (BBB) after cerebral hemorrhage can accumulate inflammatory mediators and other harmful substances within brain tissue, resulting in abnormal cerebral vascular function, including CVS. Our findings indicate reduced expression of Claudin and ZO-1 in hematoma areas compared to near-adjacent hematomas (NAH) areas, highlighting a disruption of the BBB (Figure 6A).

Next, we assessed the mRNA expression levels in previously identified mRNA-miRNA relationship pairs. In the brain tissue of patients who developed CVS following a cerebral hemorrhage, CDK6 and SLC2A1 showed upregulation in the hematoma region, where they co-localized with SMA (smooth muscle actin). However, no significant differences were detected in the expression levels of DHFR and EZH2 (Figure 6B).

Next, we treated primary human brain arterial smooth muscle cells with hemin, a key biological substance implicated in the development of CVS following subarachnoid hemorrhage (Hugelshofer et al., 2019). The results showed that the mRNA expression levels of CDK6 and DHFR initially increased after hemin treatment, followed by a decrease with prolonged exposure to hemin. After 12 h of hemin treatment, the mRNA expression of EZH2 and RB1 was downregulated. In contrast, the expression of SLC2A1 was consistently upregulated compared to the control group. We also validated the expression of miRNAs. Following hemin treatment, the levels of LET-7A-5P, MIR-24-3P, MIR-29A-3P, and MIR-132-3P were downregulated, with the lowest expression observed at 3 h post-treatment (Figure 6C). These results indicated that these mRNAs and miRNAs may play roles in the development of CVS and act as functional molecules related to CVS.

## Discussion

Cerebrovascular spasm, a critical complication, can result in delayed cerebral ischemia, delayed ischemic neurological deficits, and even cerebral infarction and fatality. Early diagnosis and effective treatment of CVS significantly reduce mortality in hemorrhagic patients, potentially saving lives. Previous studies have examined expression profiles involved in vasospasm induced by ICH to identify DEGs and DEMs. However, the results varied significantly due to differences in sequencing platforms and sample variations. Thus, a more reliable strategy to identify and screen biomarkers and targets for early detection of CVS is needed. To address this, we selected DEMs associated with CVS from the GEO database and constructed a miRNA-mRNA network. Additionally, core miRNAs (LET-7A-5P, MIR-24-3P, MIR-29A-3P, and MIR-132-3P) and their target mRNAs (CDK6 and SLC2A1) were screened from the network and validated in patients and *in vitro.* These findings suggest that these miRNAs and their target mRNAs may be involved in the regulatory network of CVS. Identifying and analyzing these core molecules can unveil the potential pathogenesis of CVS and improve the accuracy of diagnosis and treatment.

Various factors contribute to CVS, with cerebrovascular smooth muscle dysfunction being a primary cause. These factors include red blood cells and their breakdown products, impaired endothelial cell function, endothelin-1 (ET-1), nitric oxide (NO), and inflammatory mediators. The diverse inducing factors and mechanisms of CVS ultimately lead to brain tissue death. Histological staining revealed higher HE intensity in hematoma areas than in NAH, where neurons showed swelling. TUNEL and caspase-3 staining in hematoma areas indicated significant neuronal injury, apoptosis, and degeneration during intracerebral hemorrhage stages. Advances in proteomic technologies have identified potential biomarkers in brain injury and CVS, including S100β, GFAP, neurofilament proteins, APP, and tau (Tobieson et al., 2021; Batista et al., 2023). Our results align with prior research indicating elevated levels of APP, S100β, and NFL proteins in regions affected by hematomas.

Through mRNA dataset analysis, we identified 183 differentially expressed genes (114 upregulated and 69 downregulated) between the vasospasm and non-vasospasm groups (GSE37924). Subsequently, we performed enrichment analyses of GO categories and KEGG pathways to comprehensively elucidate the functions and underlying mechanisms of these genes.

The GO and KEGG pathway analyses revealed that the differentially expressed genes in the vasospasm group were enriched in the pronucleus and ATPase complex categories within the cellular component (CC) category. Additionally, they were enriched in the folic acid-containing compound biosynthetic or metabolic process and pteridine-containing compound metabolic process categories within the BP category, compared to the non-vasospasm groups. Pathway enrichment analysis suggested that the mechanisms underlying cerebral vascular vasospasm might involve regulating various pathways, including the one-carbon pool by folate, folate biosynthesis, RNA degradation, oxidative phosphorylation, and PI3K-AKT-mTOR signaling pathways. The PI3K-AKT-mTOR and oxidative phosphorylation pathways were particularly significant. The PI3K-AKT signaling pathway, a classical intracellular signaling pathway, regulates various cellular functions, such as metabolism, proliferation, cell survival, growth, and angiogenesis. This pathway is also implicated in CVS (Jin et al., 2022). Administration of Simvastatin has been demonstrated to effectively modulate the expression of PI3K-AKT-eNOS in endothelial cells, reducing the incidence of CVS following brain hemorrhage (Sugawara et al., 2008). Evidence suggests that thiazolidinediones or the calcimimetic R-568 can inhibit the expression of PI3K/Akt in vascular smooth muscle cells, suppressing vasospasm occurrence (Sinagra et al., 2013; Gulec et al., 2021). The oxidative phosphorylation signaling pathway is also involved in CVS. SAH-induced vasospasm and cerebral edema can lead to cerebral ischemia, which stimulates the production of substantial reactive oxygen species (ROS), such as superoxide ions (O_2_-) and hydrogen peroxide (H_2_O_2_). Excessive ROS production can damage neurovascular units, cause neurological injury, induce endothelin production, and impair vasodilation, leading to worsened vascular spasms (Osuka et al., 2010; Xu et al., 2022; Zhang et al., 2023). Melatonin, an antioxidant, can scavenge free radicals stimulate the expression of antioxidant enzymes (such as superoxide dismutase, glutathione peroxidase, catalase, glutathione reductase, glutathione-S-transferase, heme oxygenase, and nitric oxide synthase), promote glutathione synthesis, reduce electron leakage in the mitochondrial electron transport chain, and suppress CVS, alleviating secondary brain injury (Xu et al., 2022). Another antioxidant, edaravone, has been reported to effectively improve neurofunctional deficits, protect vascular endothelium, and alleviate DCVS in patients with SAH (Munakata et al., 2009). Excessive RNA breakdown may increase intracellular ROS production, causing damage to the neurovascular unit. These research findings are consistent with our RNA-seq analysis results.

In this research, we constructed a WGCNA network to identify modules associated with CVS. Functional annotation and pathway analysis revealed that genes within these modules are involved in various biological processes, including neuroactive ligand-receptor interaction, pantothenate and Coenzyme A (CoA) biosynthesis, glycolysis, starch and sucrose metabolism, and epithelial-mesenchymal transition, among others. Within vasospasm, neurotransmitters and hormones such as vasoconstrictors like endothelin and catecholamines induce vascular smooth muscle contraction by binding to their respective receptors, thereby precipitating vasospasm (Lin et al., 2022). CoA, in addition to its role in cellular metabolism, including fatty acid synthesis and energy metabolism, appears to regulate certain neurotransmission pathways and cell signaling, influencing the contractile state of vascular smooth muscle (Indolfi et al., 2000; Kuzuya et al., 2004). ATP, generated within the glycolytic pathway, acts as a neurotransmitter that regulates the contraction and relaxation dynamics of vascular smooth muscle, thereby influencing vascular tone and hemodynamics (Leach et al., 2002). Additionally, the glycolytic pathway plays a role in other vasospasm-related factors, including lactate production. Excessive lactate levels can induce vessel acidosis, leading to vasoconstriction and spasm. Moreover, the glycolytic pathway affects intracellular redox balance, which regulates the production of vasodilators such as nitric oxide (NO), influencing the occurrence of vasospasm (Asgari et al., 2013; Xu et al., 2023). Elevated blood glucose levels may impair endothelial function and increase intracellular calcium ions in vascular smooth muscle cells, which promotes vasospasm (Matano et al., 2019).

Our study rigorously screened and identified 15 key genes implicated in CVS and vascular smooth muscle contraction. Four of these genes, Acetylcholinesterase (AChE), diacylglycerol kinase zeta (DGKZ), NLRP3 inflammasome, and Phospholipase D2 (PLD2), have been linked to smooth muscle contraction. AChE and DGKZ directly modulate smooth muscle function (Jewulski et al., 2021), while NLRP3 inflammasome contributes to endothelial dysfunction and smooth muscle cell pyroptosis (Bai et al., 2020; Pang et al., 2022), and PLD2 is involved in angiotensin generation (Parmentier et al., 2001). Among the remaining 11 genes associated with CVS, GPD1L and GPD2, both involved in glucose metabolism, stand out, suggesting a potential link between glucose metabolism disorders and CVS. However, genes such as AGL, COL1A1, HLA-DMB, PPP1R3C, SGCD, STAT4, TAGLN, TNNT1, and WAS have been less frequently reported in relation to vasospasm and warrant further investigation. Co-expression and correlation analysis revealed strong interactions among the 15 genes, highlighting their significant roles in the occurrence and progression of CVS.

PPI networks were constructed to comprehensively explore the potential interactions and functions of significant DEGs associated with CVS. The top hub genes identified were ARID1B, EZH2, HDAC2, GART, IPO5 (RANBP5), RPL23, AKAP9, DHFR, RB1, and BRD9 based on the degree method. Most key genes were closely related to vascular smooth muscle cell or vascular endothelial cell injury. ARID1B (AT-rich interactive domain-containing protein 1B) is a component of the SWI/SNF ATP-dependent chromatin remodeling complex. The SWI/SNF complex is essential for vasculogenesis and the proliferation and differentiation of vascular smooth muscle (Julian et al., 2015). Fei Fang et al. indicated that the silencing of Brg1/Brm (components of the SWI/SNF complex) can mitigate endothelial dysfunction under inflammatory conditions, thereby alleviating phenotypes in an animal model of atherosclerosis (Fang et al., 2013). EZH2, a crucial Polycomb Repressive Complex 2 (PRC2) component, catalyzes histone H3 trimethylation at lysine 27 (H3K27me3), leading to gene repression. Elevated EZH2 levels in pulmonary arterial hypertension (PAH) patients can be targeted with GSK126, demonstrating decreased PASMC proliferation and improved mitochondrial function *in vitro* (Habbout et al., 2021; Dave et al., 2023). EZH2 also influences VSMC proliferation, migration, and vascular remodeling in conditions like atherosclerosis and abdominal aortic aneurysms (Li et al., 2022; Dave et al., 2023; Liu et al., 2023). HDAC2 (Histone Deacetylase 2) is involved in vascular smooth muscle cell (VSMC) phenotypic switching, proliferation, and migration, contributing to cardiovascular diseases (Ryu et al., 2019; Chakraborty et al., 2022). It also maintains endothelial cell barrier function, promotes angiogenesis, and regulates inflammatory responses (Hori et al., 2020). BH4 (Tetrahydrobiopterin), a product of DHFR (Dihydrofolate reductase), regulates NO signaling in endothelial cells (Thoms et al., 2016). RB1 (Retinoblastoma protein 1) is a tumor suppressor gene that inhibits the progression of the cell cycle, limits the proliferation of vascular endothelial cells, and regulates the differentiation of vascular smooth muscle cells. Studies indicated that Rb1 deficiency impairs arterial vasodilation by reducing DHFR activity and NO production in aortic endothelial cells (Cao et al., 2019). These findings strongly suggest a potential link between these key genes and the occurrence of CVS.

miRNA, a stable single-stranded non-coding RNA, regulates gene expression and has been used in diagnosing, monitoring, and treating diseases like cerebral hemorrhage-induced vasospasm (Kikkawa et al., 2017; Wang et al., 2021a; Wang et al., 2021b; Zhang Y. et al., 2022; Huang et al., 2023). In this study, we identified CVS-associated DEMs and their target genes using miRTarBase, and constructed a miRNA-mRNA network based on the negative correlation between miRNA and mRNA expression levels. Through validation with clinical specimens and *in vitro* cell experiments, we identified several miRNAs (LET-7A-5P, MIR-24-3P, MIR-29A-3P, and MIR-132-3P) and genes (CDK6 and SLC2A1) that may play significant roles in the occurrence and development of CVS. MIR-132-3P, for instance, has been shown to suppress pulmonary artery smooth muscle cell proliferation and migration after pulmonary embolism (Zhang Y. et al., 2022). Its target gene CDK6, a core component regulating the cell cycle, is considered a mutation target in malignant tumors (Meng et al., 2004).

Additionally, CDK6 has been implicated in endothelial and smooth muscle cell proliferation. CDKs were upregulated in the pulmonary vasculature of individuals with pulmonary arterial hypertension (PAH) and in animal models. Remarkably, dinaciclib and palbociclib effectively block cell cycle progression in primary pulmonary smooth muscle and endothelial cells (Weiss et al., 2019). MiR-132-3P regulates the transformation between contractile and synthetic vascular smooth muscle cells (VSMCs) (Zhang J. et al., 2022). As a target gene of miR-132-3P, SLC2A1 (GLUT1) is crucial for maintaining cellular energy supply and is closely associated with cancer prognosis (Liu et al., 2022). In various brain diseases, including malignant brain tumors, cerebral blood vessels show dysregulated expression of GLUT1 and certain junctional proteins. GLUT1 is expressed in the vascular endothelial cells of the BBB, and knocking down GLUT1, specifically in endothelial cells, increases BBB permeability, leading to vasogenic brain edema (Zheng et al., 2010). Studies on Alzheimer’s disease have shown that mice with BBB-specific GLUT1 deficiency exhibit reduced cerebral microvascular degeneration, decreased cerebral blood flow, and accelerated APP deposition (Winkler et al., 2015). Our experimental findings and these studies suggest that abnormal GLUT1 expression in cerebral arterial smooth muscle cells is implicated in the pathogenesis of cerebrovascular diseases. MiR-24-3P and MiR-29A-3P are downregulated in VSMCs and regulate abnormal proliferation induced by hypoxia and atherosclerosis (You et al., 2020; Lee and Kang, 2023; Ji et al., 2024). MiR-24-3p inhibits VSMC proliferation and promotes phenotypic transformation in atherosclerosis. Administration of miR-29a-3p also alleviates pulmonary vascular remodeling, reducing pulmonary artery pressure (Luo et al., 2015).

Therefore, combining these previous studies with our validations, we infer that these four miRNAs and two genes could serve as recovery biomarkers for patients with CVS. However, certain limitations should be noted. The sample size was relatively small, and further validation of the selected miRNA and mRNA candidates is needed to confirm their universality and reliability across larger clinical samples. Additionally, the mechanisms by which miRNA-target gene regulatory networks influence the function and contraction of vascular endothelial and smooth muscle cells require further investigation and validation.

## Conclusion

In summary, our study identified 183 DEGs and 19 DEMs associated with CVS following early diagnosis of intracranial hemorrhage. Through miRNA-mRNA network analysis, we elucidated the intricate and interdependent regulatory relationships between these critical miRNAs and their corresponding target genes, contributing to CVS development. Subsequent experimental verification confirmed the upregulation of two genes (CDK6 and SLC2A1) and the downregulation of four miRNAs (LET-7A-5P, MIR-24-3P, MIR-29A-3P, and MIR-132-3P) among the total of 6 DEGs and 8 DEMs. These results suggest a potential correlation between these genes and miRNAs, indicating their possible interplay in the development of CVS. The identified biomarkers are promising tools for the diagnosis of CVS, providing valuable insights for future research into its underlying mechanisms.

## Data Availability

The datasets presented in this study can be found in online repositories. The names of the repository/repositories and accession number(s) can be found below: https://www.ncbi.nlm.nih.gov/, GSE37924 and GSE165608.

## Abbreviations

CVS, cerebral vasospasm; DEMs, differentially expressed miRNAs; DEGs, differentially expressed genes; PPI, protein-protein interaction; ICH, intracranial hemorrhage; SAH, subarachnoid hemorrhage; DIND, delayed ischemic neurological deficits; ET, endothelin; CSF, cerebrospinal fluid; GO, Gene Ontology; KEGG, Kyoto Encyclopedia of Genes and Genomes; (aSAH), aneurysmal subarachnoid hemorrhage; WGCNA, Weighted Gene Co-expression Network Analysis; TOM, topological overlap matrix; HE, hematoxylin and eosin; ANOVA, A one-way analysis of variance; DCV, delayed cerebral vasospasm; CoA, Coenzyme A; AChE, Acetylcholinesterase; DGKZ, diacylglycerol kinase zeta; PLD2, Phospholipase D2.
