# Supplementary material for: Deciphering the role of miRNA-mRNA interactions in cerebral vasospasm post intracranial hemorrhage
Source: Front Mol Biosci. 2025 Feb 6;12:1492729. doi: 10.3389/fmolb.2025.1492729 (PMC11840915; doi:10.3389/fmolb.2025.1492729)
Supplement: Supplementary file 1 [file DataSheet2.pdf]

Supplementary Table 10. miRNA-target interactions (predicted by miRanda)

1. CDK6：hsa-let-7a-5p

<table>
<tr><th>ID</th><th>Duplex structure</th><th>Position</th><th>Score</th><th>MFE</th></tr>
<tr><td>1</td><td>miRNA 3' uuGA-UAUGUU-GGAUGAUGGAgu 5'
|| ||:||: ||| ||||||
Target 5' ctCTAATGCAGTCCTTCTACCTgg 3'</td><td>1295 - 1318</td><td>148.00</td><td>-16.80</td></tr>
<tr><td>2</td><td>miRNA 3' uugAUAUGUUGGAU-GAUGGAGu 5'
||| || |:|: |||:|||
Target 5' aagTATCCATCTTGTCTATCTCc 3'</td><td>1104 - 1126</td><td>147.00</td><td>-10.80</td></tr>
<tr><td>3</td><td>miRNA 3' uugauaUGUUGG-AUGAUGGAgu 5'
| |||| | ||||||
Target 5' aaagcaAGAACCTTCCTACCTat 3'</td><td>9320 - 9342</td><td>136.00</td><td>-13.40</td></tr>
</table>

2. DHFR：hsa-miR-24-3p

<table>
<tr><th>ID</th><th>Duplex structure</th><th>Position</th><th>Score</th><th>MFE</th></tr>
<tr><td>1</td><td>miRNA 3' gacaaGGACGACUUGACUCGgu 5'
||| | ||||||||
Target 5' catacCCT--TCAACTGAGCag 3'</td><td>327 - 346</td><td>137.00</td><td>-12.60</td></tr>
<tr><td>2</td><td>miRNA 3' gacaaggacgACUUGACUCGGu 5'
| ||:||||||
Target 5' gatggaagaaTTAATTGAGCCc 3'</td><td>1627 - 1648</td><td>136.00</td><td>-10.40</td></tr>
<tr><td>3</td><td>miRNA 3' gacaaggacgacUUGACUCGGu 5'
|||||||:|
Target 5' ctccaagaccccAACTGAGTCc 3'</td><td>188 - 209</td><td>134.00</td><td>-11.40</td></tr>
</table>

3. EZH2：hsa-let-7a-5p

<table>
<tr><th>ID</th><th>Duplex structure</th><th>Position</th><th>Score</th><th>MFE</th></tr>
<tr><td>1</td><td>miRNA 3' uugauauguUGGAUGAUGGAGu 5'
|:||:|||||||
Target 5' --------cATCTGCTACCTCc 3'</td><td>1 - 14</td><td>157.00</td><td>-16.50</td></tr>
<tr><td>2</td><td>miRNA 3' uuGAUAUGUUGGAUGAUGGAGu 5'
|| |: || | :||:|||:
Target 5' tcCTCTG-AAACAGCTGCCTTa 3'</td><td>22 - 42</td><td>123.00</td><td>-12.30</td></tr>
<tr><td>3</td><td>miRNA 3' uugaUAUGUUG---GAUGAUGGAGu 5'
|| |||| :||:|:|||:
Target 5' aaaaAT-CAACTTTTTATTGCCTTc 3'</td><td>146 - 169</td><td>113.00</td><td>-11.00</td></tr>
</table>

4. SLC2A1：hsa-miR-132-3p

5. NFIA：hsa-miR-29a-3p

<table>
<tr><th>ID</th><th>Duplex structure</th><th>Position</th><th>Score</th><th>MFE</th></tr>
<tr><td>1</td><td>miRNA 3' auuggcUAA--AGUCUACCACGAu 5'
||| ||| ||||||||
Target 5' gttgtaATTTCTCATATGGTGCTg 3'</td><td>224 - 247</td><td>162.00</td><td>-14.30</td></tr>
<tr><td>2</td><td>miRNA 3' auUGGCUAAAGUCUACCACGAu 5'
|:| ||| | ||||||||
Target 5' aaATCCATT--ACATGGTGCTa 3'</td><td>5082 - 5101</td><td>160.00</td><td>-13.50</td></tr>
<tr><td>3</td><td>miRNA 3' auUGGCUAAAGUCUACCACGAu 5'
| | | | :| ||||||||
Target 5' aaAACAAATCTA-ATGGTGCTt 3'</td><td>1071 - 1091</td><td>151.00</td><td>-12.40</td></tr>
</table>

6. NFIA：hsa-miR-223-3p

<table>
<tr><th>ID</th><th>Duplex structure</th><th>Position</th><th>Score</th><th>MFE</th></tr>
<tr><td>1</td><td>miRNA 3' acCCCAUAAACUGUUUGACUgu 5'
||| || : ||:||||
Target 5' gcGGGGATAACGAAAGCTGAgt 3'</td><td>2523 - 2544</td><td>120.00</td><td>-12.16</td></tr>
<tr><td>2</td><td>miRNA 3' accccAUAAACU--GU------UUG-ACUGu 5'
| ||||| || ||| ||||
Target 5' gccctTCTTTGAGCCAGACGCCAACTTGACc 3'</td><td>3120 - 3150</td><td>117.00</td><td>-7.10</td></tr>
<tr><td>3</td><td>miRNA 3' accccauaaACUGUUUGACUGu 5'
|| ::||| |||
Target 5' ccctgaccgTGCTGAACAGACc 3'</td><td>3534 - 3555</td><td>117.00</td><td>-6.70</td></tr>
</table>

7. RB1：hsa-miR-221-3p

<table>
<tr><th>ID</th><th>Duplex structure</th><th>Position</th><th>Score</th><th>MFE</th></tr>
<tr><td>1</td><td>miRNA 3' cuUUGGGUCG------UCUGUUACAUCGa 5'
||:|| || | | : ||||||
Target 5' taAATCCTGCCATTTAAAAAGTTGTAGCa 3'</td><td>216 - 244</td><td>123.00</td><td>-11.00</td></tr>
<tr><td>2</td><td>miRNA 3' cuUUGGGUCGUCU----GUUACAUCGa 5'
|| :|| :|| :|| |||||
Target 5' ttAAAATAGGGGATATTTAAGGTAGCt 3'</td><td>1346 - 1372</td><td>123.00</td><td>-12.90</td></tr>
<tr><td>3</td><td>miRNA 3' cuUUG-GGUCGUCUGUUACAUCGa 5'
||| || :||| |||||: |
Target 5' tgAACACCCTTAGAAAATGTGTCc 3'</td><td>419 - 442</td><td>121.00</td><td>-13.00</td></tr>
</table>

8. RB1：hsa-miR-20a-5p

<table>
<tr><th>ID</th><th>Duplex structure</th><th>Position</th><th>Score</th><th>MFE</th></tr>
<tr><td>1</td><td>miRNA 3' gaUG-GACGUGAUA---UU--CGUGAAAu 5'
|| | |:| ||| || |||||||
Target 5' taACACAGTA-TATCCCAAGTGCACTTTc 3'</td><td>863 - 890</td><td>150.00</td><td>-8.20</td></tr>
<tr><td>2</td><td>miRNA 3' gaUGGACGUGAUAUU------CGUGAAau 5'
| |||||:|| || ||:|||
Target 5' acAGCTGCATTAGAAAAAGAGGCGCTTct 3'</td><td>1248 - 1276</td><td>131.00</td><td>-16.10</td></tr>
<tr><td>3</td><td>miRNA 3' gaUGGACGUGAUAUUCGUGAAAu 5'
:|:| :| | || ||||||
Target 5' taGCTTTTAGGAAAATCACTTTg 3'</td><td>1378 - 1400</td><td>129.00</td><td>-6.20</td></tr>
</table>

9. RB1：hsa-miR-17-5p

<table>
<tr><th>ID</th><th>Duplex structure</th><th>Position</th><th>Score</th><th>MFE</th></tr>
<tr><td>1</td><td>miRNA 3' gauGGA-CGUGACA-------UU--CGUGAAAc 5'
||| ||| || || |||||||
Target 5' gacCCTAACACAGTATATCCCAAGTGCACTTTc 3'</td><td>858 - 890</td><td>146.00</td><td>-9.90</td></tr>
<tr><td>2</td><td>miRNA 3' gaUGGACGUGA-CAUU-----CGUGAAac 5'
| |||||:| | || ||:|||
Target 5' acAGCTGCATTAGAAAAAGAGGCGCTTct 3'</td><td>1248 - 1276</td><td>125.00</td><td>-13.70</td></tr>
<tr><td>3</td><td>miRNA 3' gaUGGACGUGACAUUCGUGAAAc 5'
:|:| :| || ||||||
Target 5' taGCTTTTAGGAAAATCACTTTg 3'</td><td>1378 - 1400</td><td>121.00</td><td>-8.40</td></tr>
</table>
