# Supplementary material for: Deciphering the role of miRNA-mRNA interactions in cerebral vasospasm post intracranial hemorrhage
Source: Front Mol Biosci. 2025 Feb 6;12:1492729. doi: 10.3389/fmolb.2025.1492729 (PMC11840915; doi:10.3389/fmolb.2025.1492729)
Supplement: Supplementary file 2 [file Image1.pdf]

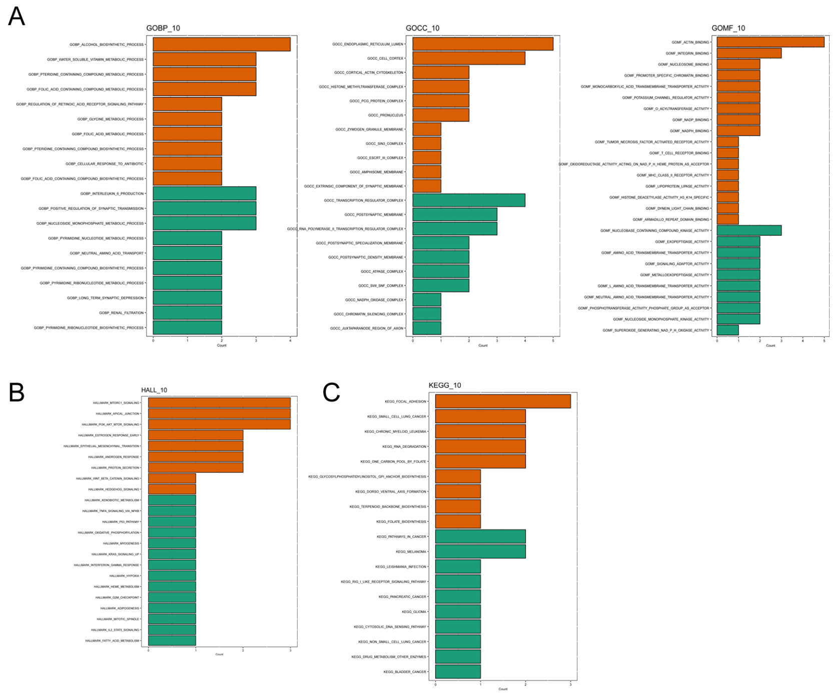


**Supplementary Figure1. Enrichment distribution plot of DEGs in GO functional categories and KEGG pathways**

(A) Enrichment analysis of Gene Ontology (GO) terms for differentially expressed genes in Biological Processes (BP), Cellular Components (CC), and Molecular Functions (MF). The X-axis represents the number of genes in count, The Y-axis represents the secondary functional categories within the primary categories of GO. The plot showing the results of the top 10 significantly enriched categories. (B-C) Hallmark enrichment analysis and KEGG pathway analysis of DEGs between vasospasm and non-vasospasm groups. The graph shows the top 10 enriched pathways. The X-axis represents the number of genes in count and Y-axis represents the description of the KEGG pathways.
